# Effects of problem-based learning on EFL learning: A systematic review

**DOI:** 10.1371/journal.pone.0307819

**Published:** 2024-12-12

**Authors:** Qian Guo, Halimah Jamil, Lilliati Ismail, Shujie Luo, Zhubin Sun

**Affiliations:** 1 Universiti Putra Malaysia, Serdang, Malaysia; 2 Chongqing Vocational and Technical University of Mechatronics, Universiti Putra Malaysia, Serdang, Malaysia; 3 Jinzhong College of Information, Jinzhong, China; Ankara University Faculty of Medicine: Ankara Universitesi Tip Fakultesi, TÜRKIYE

## Abstract

Teaching English as a foreign language (EFL) is a priority globally, but pedagogical methods do not always keep up with the evolving needs of learners. Problem-based learning (PBL) is an innovative pedagogical approach that facilitates students’ self-regulated learning, thereby improving their English proficiency. The present systematic literature review therefore concentrates on the application of PBL methodology in improving students’ English language proficiency. It was conducted according to the systematic review and meta-analysis Preferred Reporting Items for Meta-Analyses (PRISMA) review methodology. In total, 27 articles related to PBL to improve English proficiency published between 2012 and 2023 were identified from Web of Science, Scopus, ProQuest, ERIC, and ScienceDirect databases. In the light of the findings, PBL has a positive effect on students’ behaviour, academic performance, and critical thinking. Consequently, this paper contributes to policy makers, educators, and students to improve the English proficiency of students at all levels of education using PBL approach.

## Introduction

It is undeniable that the spread and dominance of English owes much to its presence in both the ‘inner circle’ (countries where English is traditionally spoken, such as Australia, the United Kingdom, and the United States) and the ‘outer circle’ (countries where English plays an integral role despite not being the mother tongue) [1]. However, it is the rapid spread of English in the ‘Expanding Circles’ (countries where English is learnt as a foreign language) that has truly established English as a global lingua franca [2, 3]. Since the late 20th century, driven by globalization and the growing mobility of business, travel, and employment worldwide, the number of non-native English speakers (NNES) has significantly exceeded the count of native English speakers (NES). An increasing number of non-native English speakers across the world are employing English as a bridge for communication among themselves, marking a significant shift from the past [4].

Nowadays, Chinese English classrooms tend to adopt teacher-driven teaching methods that focus on delivering predefined grammar, reading and writing content [5]. Such traditional English classrooms are recognized as having limited opportunities for peer and teacher interaction, relying on rote memorization and copying exercises, and emphasizing discipline and compliance with the teacher’s predetermined learning objectives and outcomes [5].

With the ongoing development of education, educators have been searching for more effective teaching methods. Students are expected to demonstrate problem-solving skills, critical thinking, and the ability to apply prior knowledge in new situations in an educational setting. Moreover, compared to traditional teacher-centred teaching methods, student-centred teaching methods offer more opportunities to improve learning outcomes, transforming the learner from a passive recipient of knowledge to an active participant in the learning process. Accordingly, it is essential to initiate pedagogical reforms in China, especially for low-performing students who are negatively affected by inappropriate teaching methods.

The PBL approach is an educational pedagogy that fits well with these expectations. Originally developed in Canadian medical schools in the 1960s as a framework for instructional design principles, the implementation of the approach has expanded to a variety of educational fields [6–8]. In particular, PBL is a constructivist approach to education that is applicable to multi-level education and is expected to be the pedagogy of choice for EFL learners [9]. The characteristics and teaching strategies of this pedagogy help learners to gain real language learning experiences and relevant information [9] Within the PBL classroom, open-ended questions are presented that are carefully designed to match the intended learning outcomes.

While PBL is a widely studied and popular method, empirical research specifically addressing its effects on the English language proficiency of EFL students remains relatively limited. Although numerous studies have investigated various aspects of PBL, there is a need for more focused research in the context of EFL education. Therefore, the purpose of this systematic literature review is to synthesize the findings related to the effects of the PBL approach on EFL learning. The objective of this paper may also serve as a guide for policy makers, educators and students to understand the effects and relationships of the PBL approach on EFL learning. This systematic literature review aims to answer the following research questions:

1. what are the effects of the PBL method on EFL learning?
2. what are the challenges of the PBL method on EFL learning?

## Methodology

This systematic review was conducted between October 2022 and February 2024 using the following protocol: search strategies and eligibility criteria, screening eligible studies, and conducting analytical studies, assessing quality and extracting data. For this systematic literature review the Preferred Reporting Items for Systematic Reviews and Meta-Analyses 2020 (PRISMA) checklist was met. This report comprises a checklist of 27 items and a four-stage flowchart for reviewing and analyzing articles [10].

## Search strategy

The search terms were categorized into two domains: a) problem-based learning; and b) EFL (see Table 1 for details of each search term). To ensure comprehensive coverage and capture the latest developments and trends in PBL pedagogy within the EFL field, a systematic review was conducted using several databases: Web of Science, Scopus, ProQuest, ERIC, and ScienceDirect. These databases were selected for their extensive collections of academic and peer-reviewed literature across diverse disciplines. The search strategy employed Boolean operations (AND, OR) to systematically explore titles, abstracts, and keywords. The search terms included "Problem-Based Learning" or "PBL" combined with "EFL," "ESL," or "English." The search was limited to articles published between 2012 and 2023 to ensure the inclusion of the most recent developments and trends. Initially, 527 results were retrieved, which were then refined to 459 results after removing duplicates.

**Table 1:** Search terms.

Domains	Search terms
Problem-based learning	"Problem-based learning" or PBL
	AND
EFL	EFL or ESL or English

In addition, the inclusion and exclusion criteria suggested by Gough, Oliver and Thomas [11] were used to avoid selection bias and make sure irrelevant studies were excluded (see Table 2).

**Table 2:** Inclusion and exclusion criteria by Gough, Oliver, & Thomas [11].

Inclusion Criteria	Exclusion Criteria
1. Published in 2012–2023	Published before 2012
2. English language	Not in English
3. EFL students’ English Learning	Participants are not in EFL context
4. Indexed in Web of Science, Scopus, and ProQuest, Eric, ScienceDirect	Not a journal article
5. The effectiveness of PBL on English learning	Not the design of PBL
6. Primary study	Not primary study like reviews

## Study selection

**Fig 1:**
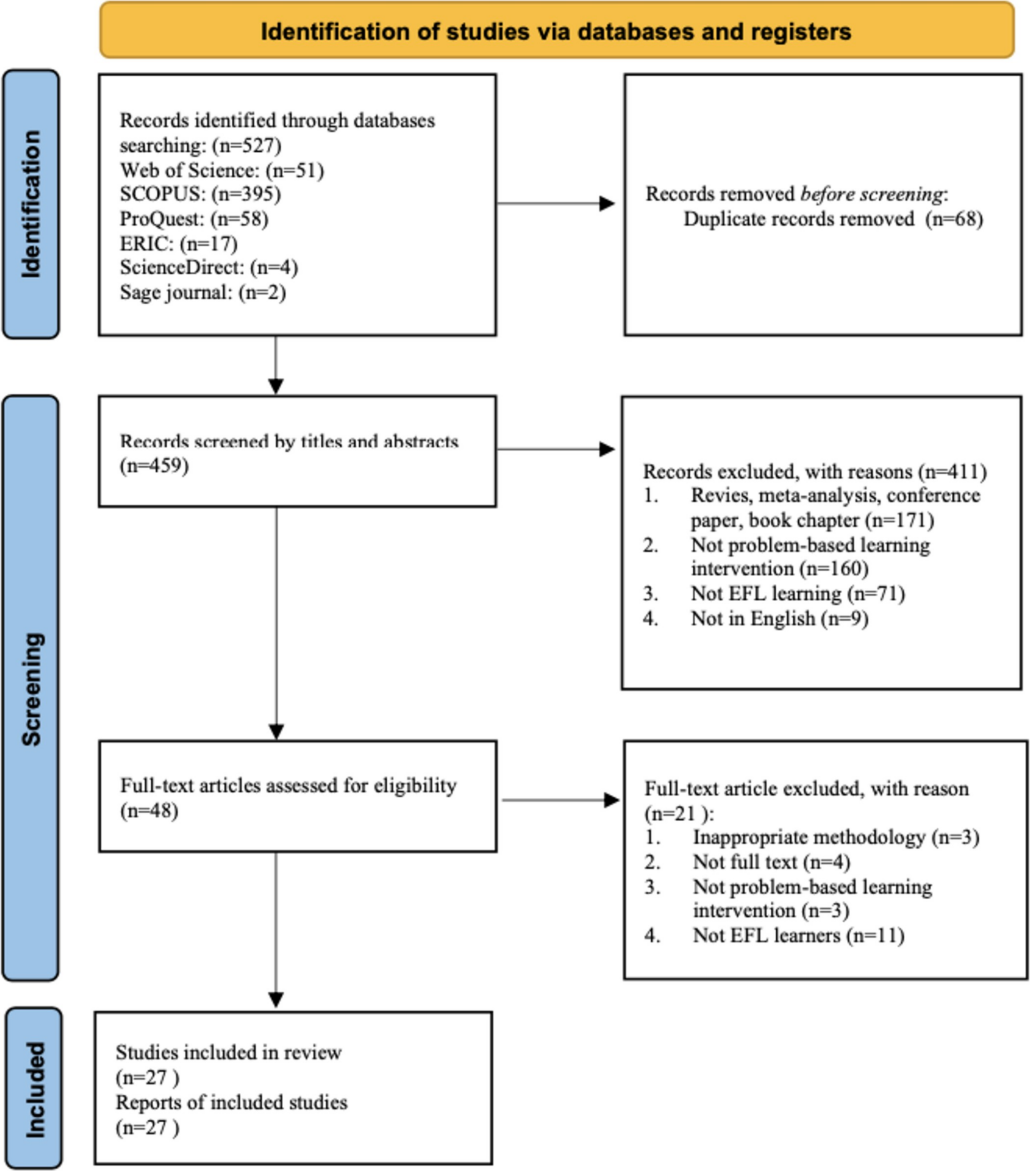
PRISMA flowchart.

Three researchers [GQ, LSJ, SZB] were responsible for title, abstract and full-text screening and quality assessment. Firstly, three researchers screened the titles and abstracts independently; if there was disagreement between them, discussions were held to reach consensus. The process of quality assessment and full paper review was the same. Fig 1 illustrates the details of the selection procedure.

## Data extraction

A total of 27 articles were included in the final stage based on the PRISMA flowchart. The information from these studies has been coded using the Population, Intervention, Comparison, Outcome and Type of Study (PICOs) tool. Two reviewers (GQ, LSJ) extracted the data for each study using Microsoft Excel spreadsheets and a third reviewer (SZB) validated the data. The data considered included 1) first author’s name and year of publication; 2) subject characteristics: sample size, gender, age, etc.; 3) characteristics of the PBL intervention, including duration (weeks), etc.; 4) characteristics of the control group, with or without a control group; 5) experimental results; 6) experimental design.

## Quality assessment

**Fig 2:**
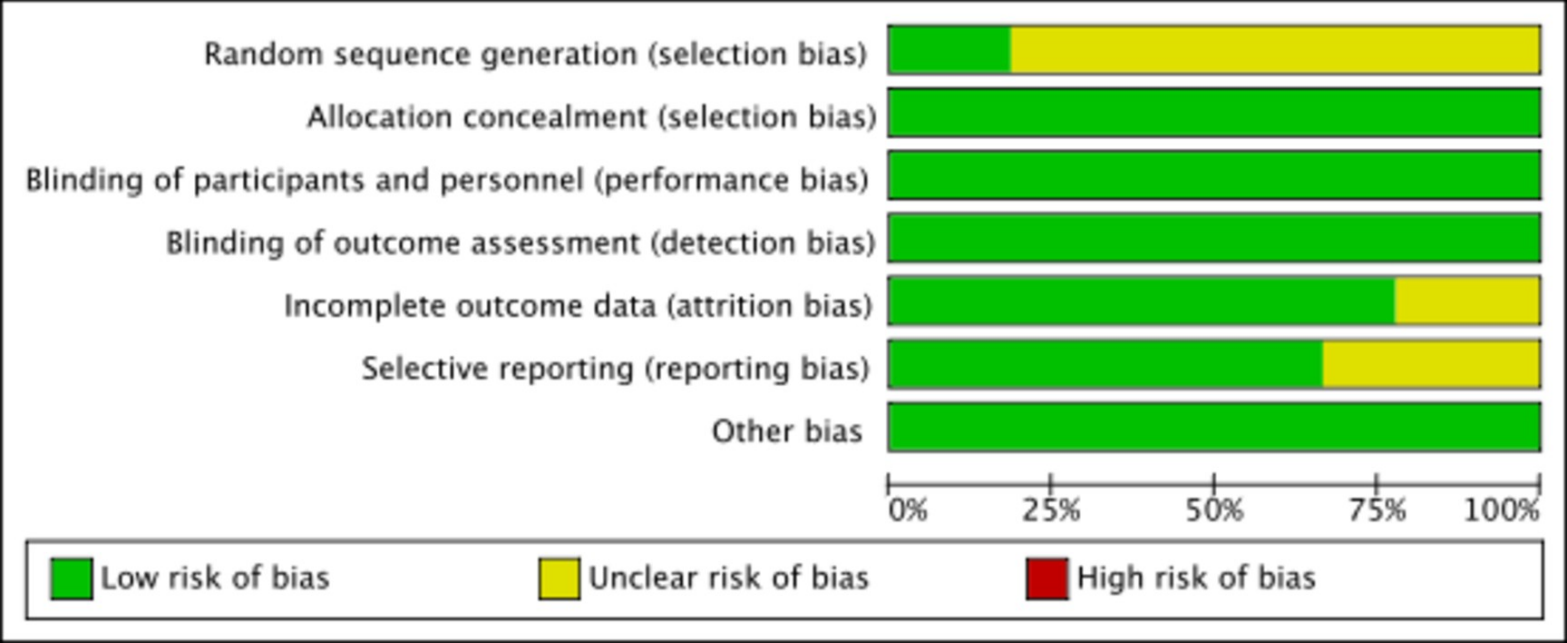
Risk of bias graph.

**Fig 3:**
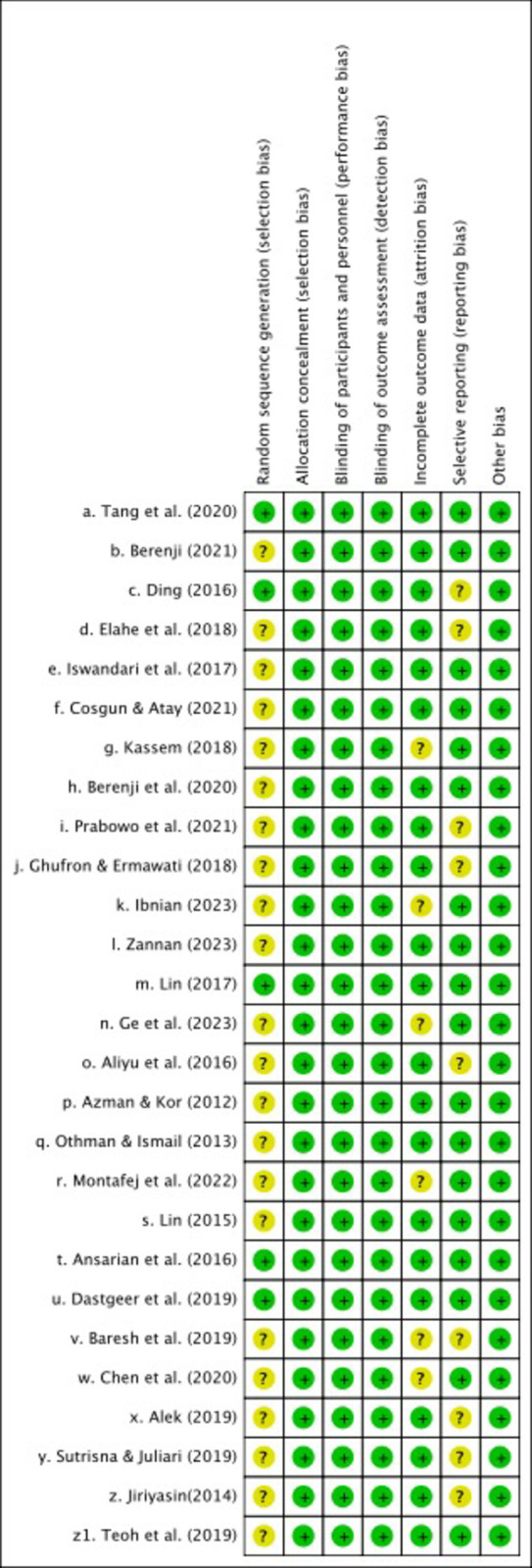
Risk of bias summary.

Two reviewers (GQ, LSJ) independently assessed the risk of bias for each identified RCT using the latest Cochrane Risk of Bias Assessment for Randomized Trials (RoB-2) [12] in accordance with the guidance on the Cochrane Training webpage. Risk Of Bias In Non-randomized Research of Interventions (ROBINS-I) was used to assess the risk of bias in non-randomized controlled trials [12]. The credibility of the evidence was analyzed and summarized using GRADE following the principles of the GRADE manual [13]. To determine the credibility of the evidence, we considered the following six factors: study design, study limitations, inconsistency, indirectness, imprecision and publication bias. Overall, the certainty of the evidence is considered to be very low to low (see Figs 2 and 3).

## Findings and discussions

Upon coding the different themes, the three main aspects appeared to measure the effectiveness of PBL pedagogy in English language teaching: student behaviour, academic achievement and critical thinking. Table 3 shows an overview of the Population, Intervention, Comparison, Outcome and Type of Study (PICOs) in the 27 studies.

**Table 3:** Overview of 27 articles.

No.	Study	P	I	C	O	S
**1**	Tang et al. (2020) [14]	freshmen; China; N = 57	PBL	traditional approach	PBL did not lead to a significant change in the teacher’s pedagogical behaviors and did not succeed in enhancing their proficiency in expressive English language.	quanti
**2**	Berenji (2021) [15]	undergraduates; Iran; N = 120	PBL	lecture-based teaching	PBL group had higher metacognitive reading strategies awareness and enhanced their comprehension ability to a high extent.	quasi
**3**	Ding (2016) [16]	undergraduates; China; N = 342	PBL (18 weeks)	/ no control group	PBL practice significantly improved EFL students’ CT disposition in general.	mixed
**4**	Elahe et al. (2018) [17]	adult EFL learners; Iran; N = 52	PBL	conventional approach	PBL can positively influence vocabulary acquisition among Iranian EFL learners.	mixed
**5**	Iswandari et al. (2017) [18]	students; Indonesia; N = 60	PBL	taught conventionally	PBL significantly enhanced their mastery of environment-related vocabulary and their writing skills.	quasi
**6**	Cosgun & Atay (2021) [19]	tertiary students; Turkey; N = 68	PBL (12 weeks)	control group	PBL increased students’ level of critical thinking and creativity and improvement in their language scores.	mixed
**7**	Kassem (2018) [20]	freshmen; Saudi Arabia; N = 60	H-PBL	control group	H-PBL approach has a positive effect on improving the students’ speaking proficiency.	mixed
**8**	Berenji et al. (2020) [21]	undergraduates; Iran; N = 118	PBL	Lecture-based control group.	PBL group had higher engagement and more enhanced reading comprehension ability.	quasi
**9**	Prabowo et al. (2021) [22]	undergraduates; Indonesia; N = 80	PBL	/ no control group	PBL improved descriptive writing among both introverted and extroverted students.	quanti
**10**	Ghufron & Ermawati (2018) [23]	students; Indonesia; N = 62	PBL	Cooperative Learning (CL)	The study noted challenges such as the need for extra time and preparation, as well as confusion reported by some students with PBL approach.	quali
**11**	Ibnian (2023) [24]	undergraduates; Amman; N = 74	online PBL (6 weeks)	control group	PBL group outperformed the control group in different essay writing areas including content, organization, vocabulary, language use, and conventions.	quasi
**12**	Zannan (2023) [25]	male students; Saudi Arabia; N = 38	PBL	control group	PBL learners perform better in their post-tests regarding grammar skills and paragraph writing and success affects students’ motivation.	mixed
**13**	Lin (2017) [26]	undergraduates; Taiwan China; N = 60	web PBL (10 weeks)	control group	PBL group achieved significantly higher mean scores than the non-PBL group in English RC and PBL significantly enhanced the participants’ active learning.	mixed
**14**	Ge et al. (2022) [27]	students; China; N = 100	online PBL (8 weeks)	control group	PBL transformed passive learning into active exploration, fostering greater student interest in online learning.	quasi
**15**	Aliyu et al. (2016) [28]	undergraduates; Nigeria, N = 18	PBL (12 weeks)	/ no control group	PBL increased students’ awareness of metacognitive knowledge which in turn can enhance their writing proficiency.	mixed
**16**	Azman & Kor (2012) [29]	undergraduates; Malaysia; N = 32	PBL (16 weeks)	control group	The students have positive perceptions on PBL and PBL has had a positive impact on the students’ language skills particularly on their speaking skills.	quasi
**17**	Othman & Ismail (2013) [30]	undergraduates; Malaysia; N = 128	PBL (14 weeks)	control group	The PBL group showed improvements in the post-writing test.	quasi
**18**	Montafej et al. (2022) [31]	undergraduates; Iran; N = 60	Pure PBL; HPBL	control group	HPBL students had significantly higher mean scores than the PPBL students who were in turn superior to their CG counterparts in terms of productive skills and critical thinking.	quasi
**19**	Lin (2015) [32]	students; Taiwan China; N = 56	PBL (12 weeks)	control group	PBL group outperformed the non-PBL group in vocabulary in the writing task.	mixed
**20**	Ansarian et al. (2016) [33]	language learners, Iran; N = 75	PBL	control group	PBL significantly increased intermediate participants’ speaking proficiency.	true experiment
**21**	Dastgeer et al. (2019) [34]	9th grade students; Pakistan; N = 831	PBL	control group	PBL improved secondary level learners’ English essay writing.	true experiment
**22**	Baresh et al. (2019) [35]	undergraduates; Libyan; N = 30	HPBL (9 weeks)	/ no control group	HPBL improved the speaking ability of the learners in fluency, grammar, comprehension, vocabulary, confidence level, intonation skills and pronunciation.	quali (case study)
**23**	Chen et al. (2021) [36]	sophomores; Taiwan China; N = 42	PBL+ VR	control group	PBL group significantly outperformed those in the control group in terms of vocabulary acquisition, and were more motivated to learn English.	mixed
**24**	Alek (2019) [37]	undergraduates; Jakarta; N = 32	PBL (8 weeks)	/ no control group	PBL model towards EFL undergraduate students is more effective in teaching and learning of English discourse reading comprehension.	mixed
**25**	Sutrisna & Juliari (2019) [38]	7^th^ grade students; Negeri; N = 41	PBL	/ no control group	PBL made significant difference in students’ writing skills before and after the study.	mixed
**26**	Jiriyasin (2014) [39]	freshmen; Thai; N = 40	PBL	/ no control group	PBL enhanced oral English performance to be more fluent and accurate.	mixed
**27**	Lin et al. (2019) [40]	undergraduates; Malaysia; N = 37	PBL (4 months)	control group	PBL increased in their listening comprehension scores as compared to the control group participants.	mixed

### Effects of PBL on student behaviour

Six studies (Studies 1, 8, 12, 13, 14, 23) were dedicated to examining how PBL teaching methods affect learners’ learning behavior, focusing on aspects like engagement, motivation, active participation, and readiness for learning. An examination of these studies concerning the utilization of PBL in English language instruction consistently indicates its positive impact on student learning behavior and academic performance. Through diverse inquiries, PBL approaches consistently showcased beneficial outcomes in terms of enhancing student engagement, motivation, and active involvement in the learning process.

In Studies 8 and 14, researchers employed a quasi-experimental design to assess the effectiveness of PBL in various educational settings. Study 8, led by Berenji et al. [21] at a Chinese university, compared PBL, facilitated through group discussions, with traditional lecture-based teaching. Their findings revealed that PBL promoted practical learning goals by utilizing authentic questions, prompting students to utilize prior knowledge and scaffolding to overcome reading challenges. This method resulted in increased self-determination and engagement among students, contrasting with the lower engagement levels observed in the control group receiving lecture-based instruction. Similarly, in Study 14 by Ge et al. [27], the researchers examined the impact of PBL on student readiness and performance in online English learning at a Chinese high school. They discovered that integrating PBL activities into online learning significantly enhanced student readiness, especially during the shift to online education. Through the integration of online platforms and emphasis on student-centered teaching, PBL transformed passive learning into active exploration, fostering greater student interest. This resonates with theories proposed by Zuo et al. [41] and Yan et al. [42], indicating that PBL promotes a stronger psychological inclination among students to actively participate in group discussions.

Studies 12, 13, and 23 employed a mixed-method approach to examine the impact of PBL on students’ motivation and active learning. In Study 12 by Zannan [25], the focus was on the influence of PBL on students’ motivation in learning English writing. The study found that students exhibited improved performance in post-tests compared to pre-tests, indicating a positive effect of PBL on motivation. Similarly, Study 23, conducted by Chen et al. [36], investigated the motivation of 42 sophomore students in electrical and mechanical engineering in Taiwan. They were split into two groups, with one group receiving PBL and virtual reality (VR) instruction, and the other serving as the control. Results revealed that students in the experimental group were more motivated to learn English relevant to their future careers. Likewise, Study 13 utilized a questionnaire to explore the impact of PBL on participants’ active learning and cognitive processing, finding significant improvements in both aspects. By employing a combination of qualitative and quantitative methods, these studies offer comprehensive insights into the varied benefits of PBL, including enhanced motivation, active learning, and cognitive engagement among students. Such findings not only enrich the existing research on PBL effectiveness but also provide valuable guidance for educators aiming to improve student learning outcomes in English language education through innovative teaching methods.

Study 1, which also examined the impact of implementing the PBL approach on student behavior, presented contrasting findings. Despite an increased emphasis on group discussions within PBL classes, Tang et al. [14] found, through quantitative analysis, that traditional lecturing remained the dominant instructional method. These results suggest that the integration of PBL strategies in English classrooms has not substantially changed teachers’ instructional practices. This lack of change in teaching behavior could impede the intended objective of providing students with ample opportunities to improve their English expression skills through active learning methods.

These findings highlight several challenges in effectively implementing PBL approaches in English language education. One notable challenge is the resistance to change among educators, as traditional lecturing methods persist despite efforts to incorporate PBL strategies. This resistance underscores the need for comprehensive teacher training and support to facilitate the adoption of innovative teaching approaches. Additionally, there appears to be a lack of consistent implementation of PBL techniques, indicating potential gaps in teacher preparation or resource allocation. Limited resources, including time, materials, and support, further compound the challenges faced by educators in fully embracing PBL. Moreover, there may be a disconnect between the objectives of PBL and broader educational goals, highlighting the importance of aligning pedagogical approaches with curriculum requirements. Addressing these challenges requires a concerted effort from educational institutions to provide ongoing professional development opportunities, allocate sufficient resources, and ensure pedagogical alignment to enhance the effective integration of PBL into English language education.

### Effects of PBL on academic performance

In the evaluation of how PBL pedagogy influences students’ academic performance, a total of twenty-four studies were reviewed. Among these, four studies (Studies 2, 8, 13, and 24) examined reading proficiency, while nine studies (Studies 5, 9, 10, 11, 12, 15, 17, 21, and 25) focused on writing skills. Furthermore, five studies (Studies 7, 16, 20, 22, and 26) investigated speaking fluency, with only one study (Study 27) concentrating on listening skills. Additionally, vocabulary acquisition was explored in five studies (Studies 4, 5, 11, 19, and 23).

Only four studies were included in the analysis regarding reading proficiency. In Study 2, Berenji [15] conducted a quasi-experimental study comparing the effects of two teaching methods: PBL for the experimental group and lecture-based teaching for the control group, on reading proficiency skills. The results indicated that the experimental group demonstrated increased awareness of metacognitive reading strategies and significantly improved their reading proficiency. Similar results were found in Berenji et al.’s [21] Study 8, showing heightened engagement and improved reading proficiency in the PBL group. Implementing student-centered approaches like PBL could cultivate highly engaged and successful learners in reading proficiency lessons. Furthermore, Lin’s [26] Study 13 integrated PBL into a web-based English reading course and found that the PBL group achieved significantly higher adjusted mean scores on the posttest compared to the non-PBL group. Alek’s [37] study24, while demonstrating the effectiveness of PBL in improving English as a Foreign Language (EFL) undergraduate students’ English discourse reading comprehension, lacked a control group.

These findings highlight several challenges in the studies examining the impact of PBL on reading proficiency. One significant challenge is the absence of control groups in some studies, as seen in Study 24 by Alek [37]. Without control groups, it becomes difficult to establish a clear causal relationship between the implementation of PBL and observed improvements in reading proficiency. This lack of control groups not only raises concerns about the methodological rigor of the studies but also limits their ability to attribute improvements solely to the PBL approach. Moreover, while PBL shows promise in enhancing reading proficiency and metacognitive reading strategies, the implementation of PBL in EFL contexts may pose logistical challenges, such as training educators in PBL methods and designing appropriate PBL activities tailored to the language learning context. Addressing these challenges requires careful consideration of research design, including the incorporation of control groups and the use of rigorous experimental methodologies, as well as targeted support for educators in implementing PBL effectively in language learning environments.

In nine studies, researchers investigated the impact of PBL pedagogy on students’ writing proficiency. Studies 15 and 25 utilized a mixed design methodology to explore the effectiveness of PBL in diverse educational settings. In Study 15, conducted by Aliyu et al. [28] in North-Eastern Nigeria, significant improvements were noted in participants’ metacognitive knowledge across various domains, including task understanding, learning processes, strategy use, text comprehension, problem-solving, and discourse features. Interviews emphasized the role of real-life relevant problems and interactive PBL processes in enhancing metacognitive awareness, suggesting potential benefits for ESL students’ proficiency and writing skills. Similarly, Study 25 by Sutrisna and Juliari [38] involved seventh-grade students from SMP Negeri 4 Denpasar. Employing a PBL approach, the study observed a significant enhancement in students’ writing skills following the intervention. Notably, the problems presented during PBL activities stimulated students to improve both the content and organization of their writing. Both studies highlight the effectiveness of PBL in nurturing cognitive and writing skills development, supported by qualitative insights into the interactive and problem-driven nature of the learning process. In the same vein, Study 12, conducted by Zannan [25], also produced notable findings using a mixed design, with learners in the experimental group utilizing PBL showing significant improvements in grammar skills and passage writing. This indicates the efficacy of PBL in fostering English writing proficiency, suggesting a need for further exploration into the specific mechanisms through which PBL enhances writing skills.

Across several quasi-experimental studies, Iswandari et al. [18], Ibnian [24], and Othman and Ismail [30] examined the effectiveness of PBL in improving students’ writing skills. Iswandari et al. [18] discovered significant enhancements in writing abilities through environmental PBL instruction in Study 5. Ibnian [24] focused on online PBL in Study 11, where the experimental group exhibited superior performance in various aspects of essay writing compared to the control group. Othman and Ismail [30] implemented a 14-week PBL approach with a control group in Study 17, revealing that while both groups progressed in course content, the PBL group demonstrated greater advancements in language proficiency, indicating the effectiveness of PBL in fostering deeper language skill development. Collectively, these studies suggest that PBL shows promise in improving students’ writing proficiency across diverse educational settings.

In studies 21 and 9, led by Dastgeer et al. [34] and Prabowo et al. [22] respectively, the effectiveness of PBL in enhancing students’ writing skills was assessed. Dastgeer et al. [34] utilized a true experimental research design involving 9th-grade students from Islamabad Model Schools, revealing that PBL proved more effective than conventional methods in improving English essay writing among secondary-level learners. Similarly, Prabowo et al. [22] conducted quantitative research demonstrating improved descriptive writing among both introverted and extroverted students exposed to PBL. These findings underscore PBL’s role in stimulating students’ ideation processes and enhancing their writing abilities, underscoring the need for further investigation in this domain to deepen our understanding of PBL’s impact on writing proficiency.

However, it is crucial to acknowledge the potential challenges associated with implementing the PBL approach in teaching English writing, as highlighted in Study 10 by Ghfron and Ermawati [23], conducted through a qualitative research design using a case study approach. The study underscored that implementing PBL demands additional time, preparation, and effective management, presenting significant challenges for teachers. Moreover, some students expressed confusion with the PBL approach. These findings resonate with research by Leong [43], which also noted that one potential drawback of PBL is its initial tendency to confuse students. Additionally, certain students may hesitate to engage in discussions or articulate their perspectives on problem-solving tasks, while teachers may struggle with formulating open-ended questions throughout the learning process. These insights shed light on the potential hurdles educators may encounter when integrating PBL into English writing instruction. Hence, meticulous planning, adequate support, and effective strategies are essential to address these challenges and ensure the successful implementation of the PBL approach.

Five research studies have delved into teaching English speaking skills. Both Study 7 by Kassem [20] and Study 26 by Jiriyasin [39] employed a mixed research design to assess the impact of PBL on students’ oral English proficiency. In Study 7, which focused on the Hybrid Problem-Based Learning (H-PBL) method, positive outcomes emerged, including heightened learning motivation, increased autonomy in language learning, and favorable feedback from teachers. Similarly, Study 26 revealed significant enhancements in students’ oral performance subsequent to a traditional PBL intervention, characterized by improved fluency, accuracy, and the utilization of diverse sentence structures.

The studies conducted by Azman and Kor [29], Ansarian et al. [33], and et al. [35] share a common objective of improving students’ speaking skills in English through the implementation of PBL. Despite variations in research design, location, participants, type of PBL, data analysis, and outcomes, each study examined the effectiveness of PBL in enhancing speaking proficiency among students. All three studies reported positive impacts of PBL on speaking abilities, with students demonstrating improvements in fluency, grammar, comprehension, vocabulary, confidence level, intonation skills, and pronunciation.

While the studies collectively demonstrate the positive impact of PBL on students’ speaking skills in English, several challenges can be identified. Firstly, the methodological variations across the studies, such as the use of different research designs and the absence of control groups in some cases, may limit the generalizability of the findings and complicate comparisons between interventions. Additionally, the diverse implementation of PBL methods, ranging from traditional to hybrid approaches, raises questions about the optimal approach for improving speaking proficiency. Moreover, while the studies reported improvements in various aspects of speaking skills, such as fluency, grammar, and vocabulary, the extent to which these improvements translate into real-world communicative competence is unclear. Furthermore, the necessity for further research to explore the effectiveness of PBL across diverse contexts and populations suggests that more nuanced considerations, such as cultural differences and individual learner characteristics, need to be addressed to fully understand the impact of PBL on speaking skills in English.

In the domain of listening skills, Study 27 by Teoh et al. [40] stands as a solitary study focusing on the efficacy of PBL in enhancing listening comprehension skills among first-year undergraduate students. Utilizing a mixed research design, both experimental and control groups underwent the same listening test as a post-assessment following the treatment period. The results indicated a notable improvement in listening comprehension scores among students in the experimental group compared to those in the control group, suggesting the potential effectiveness of PBL in enhancing receptive language skills, particularly in listening comprehension. However, due to the limited scope of only one study addressing this area, there exists a clear need for further research. Additional investigations will provide a deeper understanding of how PBL influences students’ listening comprehension. By conducting more research in this domain, scholars can corroborate and expand upon the findings of Teoh et al. [40], ultimately advancing knowledge and informing the development of more effective educational practices in listening skill development.

In the context of the sole study focused on enhancing listening comprehension skills through PBL, several challenges can be inferred. Firstly, the limited scope of research in this area presents a challenge in comprehensively understanding the efficacy of PBL in improving listening skills. With only one study available, it is difficult to draw definitive conclusions about the generalizability and effectiveness of PBL in diverse educational settings and student populations. Moreover, incorporating PBL into listening instruction may require significant time, resources, and effective management, potentially presenting barriers for teachers. Furthermore, the effectiveness of PBL in improving listening comprehension may vary depending on factors such as student engagement, prior knowledge, and language proficiency levels, posing challenges in ensuring equitable outcomes for all students. Addressing these challenges requires further research to explore the nuances of implementing PBL in enhancing listening skills and developing targeted strategies to overcome potential barriers in its implementation.

In the realm of vocabulary acquisition, five studies have examined the impact of PBL in various educational settings. Elahe et al. [17] and Lin [32] conducted mixed-method studies, while Chen et al. [42] utilized a mixed research design, all focusing on PBL’s effect on vocabulary learning. Elahe et al. [17] demonstrated in Study 4 that PBL significantly influenced the vocabulary learning of Iranian EFL learners, with learners adopting strategies to overcome challenges encountered during the PBL process and expressing positive views towards PBL. Lin [32] conducted a 12-week PBL intervention in an elementary school in Taiwan, showing that while there were no significant differences in test scores between the PBL and control groups, the PBL group exhibited superior performance in using advanced vocabulary levels and writing longer compositions, indicating the potential of PBL to enhance vocabulary acquisition. In Study 23, Chen et al. [36] found that a PBL approach combined with virtual reality (VR) technology led to significant improvements in vocabulary acquisition and increased motivation among engineering students in Taiwan. However, the lack of significant differences in problem-solving performance raises questions about the broader impact of the intervention beyond vocabulary acquisition. Further research is necessary to explore the effectiveness of this approach in improving overall language skills and academic performance.

The positive impact of PBL on vocabulary acquisition is further supported by the quasi-experimental study conducted by Iswandari et al. [18] (Study 5), which revealed significant improvement in students’ mastery of environment-related vocabulary when taught using environmental PBL. Notably, this study innovatively combined PBL with environmental problems to assess the acquisition of environment-related vocabulary and writing skills. Similarly, in Study 11, Ibnian [24] highlighted substantial enhancements in English writing proficiency among students in the experimental group after the PBL intervention, with vocabulary utilization being one of the observed improvements.

Although the studies reviewed demonstrate the beneficial effects of PBL on vocabulary acquisition, they also highlight several challenges associated with implementing this method. Firstly, variations in research design, educational settings, and participant demographics across the studies may obscure a comprehensive understanding of the challenges inherent in implementing PBL for vocabulary acquisition. Furthermore, while PBL has shown promise in enhancing specific aspects of vocabulary learning, such as environment-related vocabulary, its effectiveness in broader vocabulary acquisition contexts or across different language proficiency levels remains uncertain. Thus, while the studies demonstrate the potential benefits of PBL in vocabulary acquisition, further research is needed to explore and address the challenges associated with its implementation in diverse educational settings and student populations.

### Effects of PBL on critical thinking

The studies conducted by Ding [16], Cosgun and Atay [19], and Montafej et al. [31] provide valuable insights into the impact of PBL on language skills, critical thinking (CT), and creativity among students. Ding [16] demonstrated in Study 3 that the implementation of PBL significantly improved the CT processing abilities of EFL students, particularly during the independent inquiry phase and across all CT subscales. However, noticeable gender differences were observed in certain aspects, such as the inclination to hypothesize after engaging in the PBL exercise. Similarly, Cosgun and Atay [19], in Study 6, also found significant enhancements in critical thinking, creativity, and language scores following the implementation of PBL. Additionally, Montafej et al. [31], in Study 18, compared the effectiveness of different PBL approaches and a Mobile Assisted Language Learning (MALL) application in improving language skills and critical thinking among Iranian undergraduates. Their findings indicated that Hybrid Problem-Based Learning (HPBL) combined with MALL applications led to significantly higher scores in productive skills and critical thinking compared to other PBL approaches and the control group. Overall, these studies offer valuable insights into the multifaceted benefits of PBL in language education and underscore its potential to promote critical thinking skills alongside language proficiency.

The studies conducted by Ding [16], Cosgun and Atay [19], and Montafej et al. [31] demonstrate the positive effects of PBL on language skills and critical thinking (CT), yet they also reveal several challenges in implementing PBL. Firstly, despite the observed improvements in CT processing abilities and language scores, Ding [16]noted noticeable gender differences in certain aspects, such as hypothesizing, following PBL exercises. This suggests that while PBL can be effective, it may not fully address underlying gender disparities in student engagement or participation. Additionally, Cosgun and Atay [19] found enhancements in critical thinking and creativity post-PBL implementation, but challenges in measuring and assessing these abstract constructs may limit the comprehensive understanding of PBL’s impact. Moreover, while Montafej et al. [31] highlighted the effectiveness of Hybrid Problem-Based Learning (HPBL) combined with Mobile Assisted Language Learning (MALL) applications, the integration of technology into PBL may pose logistical challenges, such as access to technology and technical support. Furthermore, ensuring equitable access to resources and support for all students, particularly in contexts with limited technological infrastructure, remains a challenge in implementing technology-enhanced PBL approaches. Additionally, the implementation of PBL may require substantial time, resources, and training for educators to effectively design and facilitate PBL activities, posing challenges in terms of workload and professional development. Addressing these challenges requires careful consideration and strategic planning to ensure that PBL initiatives are inclusive, effectively address gender disparities, and leverage technology to enhance student learning experiences and critical thinking skills.

## Conclusion

This systematic literature review analyzed 27 articles on the effectiveness of PBL methodology in improving English language proficiency, especially listening, speaking, reading, writing, vocabulary growth, learning behaviour and critical thinking. Out of these 27 articles, 25 studies found that PBL had a positive effect in enhancing learning behaviour, academic performance and critical thinking of EFL students. Findings indicated that the core strengths of PBL were primarily self-regulated learning, interaction, engagement, and active learning. Students take responsibility for their own learning process, cooperate with each other and share ideas, which increases opportunities for communication and interaction and facilitates active learning during language learning. There is no doubt that the PBL approach creates a more favorable learning environment for teaching English language proficiency. However, implementing a PBL pedagogy poses several challenges that need to be addressed. To begin with, resistance to change among educators, who often adhere to traditional lecturing methods, poses a significant challenge to implementing PBL strategies. This highlights the importance of providing comprehensive teacher training and support to encourage the adoption of innovative teaching approaches. In addition, when implementing the PBL pedagogy, teachers need to plan their classes carefully and clarify the process and purpose of the method to their students in advance to avoid any confusion about the method. If English teachers are not familiar with the PBL methodology, interventions will not be effective and students will be confused during the learning process. Moreover, there are practical challenges that need to be considered, such as ’’equipment’’, ’’technology’’, ’’difficulty in formulating appropriate questions/scenarios’’, ’’location of the study’’ and ’’diverse educational settings’’. There is still a long way to go in using PBL to effectively enhance the performance of EFL learners. Only when both teachers and learners are convinced of the effectiveness and relevance of PBL for a specific purpose will they be able to commit wholeheartedly to this style of teaching.

In general, the results of the study may provide useful insights for policy makers (e.g. curriculum developers) to develop more effective pedagogical approaches to guide the teaching and learning of English with greater confidence. In addition, educators, EFL and ESL teachers can benefit from the results of this study by increasing their knowledge of PBL pedagogy. The results of the study can also help textbook writers in various ways, such as selecting suitable teaching materials and optimizing the allocation of study time.

The literature review also identified several research gaps. For example, Lin et al. [40] conducted a study highlighting PBL potential effectiveness in improving listening comprehension among first-year undergraduate students. Their mixed-methods approach demonstrated significant improvement in the experimental group compared to the control group. However, this study stands alone, underscoring a notable research gap in the field. The limited breadth of existing literature necessitates further investigation to validate and build upon these initial findings. Additional research is crucial for gaining deeper insights into how PBL impacts listening comprehension, corroborating Lin et al.’s [40] findings, and advancing educational practices aimed at enhancing listening skills across different educational settings. Moreover, most existing studies primarily rely on quantitative self-reports (like surveys and questionnaires) supplemented by qualitative measures (such as interviews and discourse analyses) to explore student behaviors, including learner engagement. Only a few studies [23, 38] have incorporated classroom observations and field notes to examine the effects of PBL pedagogy on learner engagement. Future research should integrate a broader range of data collection methods, including more observational studies and field notes, to comprehensively understand how PBL influences student behaviors. Additionally, while PBL shows promise in enhancing language learning outcomes, its effectiveness may vary depending on implementation strategies and environmental factors. Further research is necessary to determine optimal approaches for applying PBL to EFL reading instruction in China, where students often struggle with reading proficiency due to traditional teaching methods. PBL offers an alternative approach, but its impact on improving reading proficiency needs thorough investigation. Overall, addressing these research gaps and methodological limitations is critical for advancing the understanding and application of PBL in EFL contexts.

### Recommendations

The PBL approach unquestionably serves as a promising model for enhancing English language proficiency. Nevertheless, it is imperative for researchers to undertake more comprehensive studies to address the issues and seek solutions to the challenges confronted by students in specific aspects of English language proficiency, such as reading in English. It is also noteworthy that none of the investigations into the impact of PBL on English reading proficiency have been conducted in mainland Chinese universities. With the exception of one study Lin [26] in Taiwan Province, China, this specific context remains largely unexplored.

### Limitations

Given the limitations of this work, more academic databases and search engines such as JSTOR, Springer, etc. should be explored. By doing so, a broader and more detailed search of the literature background related to PBL and English language proficiency should be conducted. In addition, some research articles have advocated the effectiveness of the PBL approach in developing students’ reading proficiency, hence there is a need to continue to deepen this research in order to draw solid conclusions from the implementation of this approach.

## Supporting information

S1 Checklist PRISMA 2020 checklist. (PDF)

S1 File ROB 27. (PDF)
