# Supplementary material for: Effects of problem-based learning on EFL learning: A systematic review
Source: PLoS One. 2024 Dec 12;19(12):e0307819. doi: 10.1371/journal.pone.0307819 (PMC11637334; doi:10.1371/journal.pone.0307819)
Supplement: S1 File — (PDF) [file pone.0307819.s002.pdf]

GRADE Assessment for RCTs

No. of Studies (total participants)	Design	Study Limitation	Inconsistency	Indirectness	Imprecision	Publication Bias	Certainty (overall score)
**Outcome: Student behaviour**							
a.Tang et al. (2020)<br><br>(n=57)	RCT	No downgrading	Only one study meaning inconsistency is not applicable.	Not Downgraded.	Not Downgraded.	None detected	⊕○○○ **Very low**
**Outcome: Critical thinking**							
c.Ding (2016)<br><br>(n=342)	RCT	No downgrading	Only one study meaning inconsistency is not applicable.	Not Downgraded.	Downgraded one level due to serious imprecision: no control group; 4 instructors, including the researcher herself	None detected	⊕⊕○○ **Low**
**Outcome: Student behaviour + Academic performance**							
m.Lin (2017)<br><br>(n=60)	RCT	No downgrading	Only one study meaning inconsistency is not applicable.	Not Downgraded.	Not Downgraded.	None detected	⊕○○○ **Very low**
**Outcome: Academic performance**							
t.Ansarian et al. (2016)<br><br>(n=75)	RCT	No downgrading	Only one study meaning inconsistency is not applicable.	Not Downgraded.	Not Downgraded.	None detected	⊕○○○ **Very low**
**Outcome: Academic performance**							
u.Dastgeer et al. (2019)<br><br>(n=831)	RCT	No downgrading	Only one study meaning inconsistency is not applicable.	Not Downgraded.	Not Downgraded.	None detected	⊕○○○ **Very low**

GRADE assessment for Non-RCTs

No. of Studies (total participants)	Design	Study Limitations	Inconsistency	Indirectness	Imprecision	Publication Bias	Certainty (overall score)
**Outcome: Academic performance**							
b.Berenji (2021)<br><br>(n=120)	Non-RCT	No downgrading	Only one study meaning inconsistency is not applicable.	No downgrading	Not Downgraded	None detected	⊕○○○ **Very low**
**Outcome: Academic performance**							
d.Elahe et al. (2018)<br><br>(n=52)	Non-RCTs	No downgrading	Only one study meaning inconsistency is not applicable	No downgrading	Downgraded one level due to serious imprecision: unclear sampling	None detected	⊕⊕○○ **Low**
**Outcome: Academic performance**							
e.Iswandari et al. (2017)<br><br>(n=60)	Non-RCTs	No downgrading	Only one study meaning inconsistency is not applicable	No downgrading	Not Downgraded	None detected	⊕○○○ **Very low**
**Outcome: Critical thinking**							
f.Cosgun & Atay (2021)<br><br>(n=68)	Non-RCT	No downgrading	Only one study meaning inconsistency is not applicable	No downgrading	Not Downgraded	None detected	⊕○○○ **Very low**
**Outcome: Academic performance**							
g.Kassem (2018)<br><br>(n=60)	Non-RCTs	No downgrading	Only one study meaning inconsistency is not applicable	Downgraded one level due to serious Indirectness: H-PBL not PBL	Not Downgraded	None detected	⊕⊕○○ **Low**
**Outcome: Student behaviour + Academic performance**							
h.Berenji et al. (2020)<br>(n=118)	Non-RCTs	No downgrading	Only one study meaning inconsistency is not applicable	No downgrading	Not Downgraded	None detected	⊕○○○ **Very low**
**Outcome: Academic performance**							
i.Prabowo et al. (2021)<br><br>(n=80)	Non-RCT	No downgrading	Only one study meaning inconsistency is not applicable.	No downgrading	Downgraded one level due to serious imprecision: no control group	None detected	⊕⊕○○ **Low**
**Outcome: Academic performance**							
j.Ghfron & Ermawati (2018)<br><br>(n=62)	Non-RCT	No downgrading	Only one study meaning inconsistency is not applicable	No downgrading	Downgraded one level due to serious imprecision: 2 instructor	None detected	⊕⊕○○ **Low**
**Outcome: Academic performance**							

k.Ibnian (2023)<br><br>(n=74)	Non-RCT	No downgrading	Only one study meaning inconsistency is not applicable	Downgraded one level due to serious Indirectness: online PBL not PBL	Not Downgraded	None detected	⊕⊕○○ **Low**
**Outcome: Student behaviour**							
l. Zannan (2023)<br><br>(n=38)	Non-RCT	No downgrading	Only one study meaning inconsistency is not applicable	No downgrading	Not Downgraded	None detected	⊕○○○ **Very low**
**Outcome: Student behaviour**							
n.Ge et al., (2023)<br><br>(n=100)	Non-RCT	No downgrading	Only one study meaning inconsistency is not applicable	Downgraded one level due to serious Indirectness: integrating with online learning	Not Downgraded	None detected	⊕⊕○○ **Low**
**Outcome: Academic performance**							
o.Aliyu et al. (2016)<br><br>(n=18)	Non-RCT	No downgrading	Only one study meaning inconsistency is not applicable	No downgrading	Downgraded one level due to serious imprecision: no control group; small sampling	None detected	⊕⊕○○ **Low**
**Outcome: Academic performance**							
p.Azman & Kor (2012)<br><br>(n=32)	Non-RCT	No downgrading	Only one study meaning inconsistency is not applicable	No downgrading	Not Downgraded	None detected	⊕○○○ **Very low**
**Outcome: Academic performance**							
q.Othman & Ismail (2013)<br><br>(n=128)	Non-RCT	No downgrading	Only one study meaning inconsistency is not applicable	No downgrading	Not Downgraded	None detected	⊕○○○ **Very low**
**Outcome: Critical thinking**							
r. Montafej et al. (2022)<br><br>(n=60)	Non-RCT	No downgrading	Only one study meaning inconsistency is not applicable	Downgraded one level due to serious Indirectness: Pure PBL and HPBL not PBL	Not Downgraded	None detected	⊕⊕○○ **Low**
**Outcome: Academic performance**							
s. Lin (2015)<br><br>(n=56)	Non-RCT	No downgrading	Only one study meaning inconsistency is not applicable	No downgrading	Not Downgraded	None detected	⊕○○○ **Very low**
**Outcome: Academic performance**							
v. Baresh et al. (2019)<br><br>(n=30)	Non-RCT	No downgrading	Only one study meaning inconsistency is not applicable	Downgraded one level due to serious Indirectness: HPBL not PBL	Downgraded one level due to serious imprecision: no control group;	None detected	⊕⊕○○ **Low**
**Outcome: Student behaviour**							

w. Chen et al. (2020)<br><br>(n=42)	Non-RCT	No downgrading	Only one study meaning inconsistency is not applicable	Downgraded one level due to serious Indirectness: PBL+VR not PBL	Not Downgraded	None detected	⊕○○○ **Very low**
**Outcome: Academic performance**							
x. Alek (2019)<br><br>(n=32)	Non-RCT	No downgrading	Only one study meaning inconsistency is not applicable	No downgrading	Downgraded one level due to serious imprecision: no control group;	None detected	⊕⊕○○ **Low**
**Outcome: Academic performance**							
y. Sutrisna & Juliari (2019)<br><br>(n=41)	Non-RCT	No downgrading	Only one study meaning inconsistency is not applicable	No downgrading	Downgraded one level due to serious imprecision: no control group;	None detected	⊕⊕○○ **Low**
**Outcome: Academic performance**							
z. Jiriyasin (2014)<br><br>(n=40)	Non-RCT	No downgrading	Only one study meaning inconsistency is not applicable	No downgrading	Downgraded one level due to serious imprecision: no control group;	None detected	⊕⊕○○ **Low**
**Outcome: Academic performance**							
z1.Teoh et al.(2019)<br><br>(n=37)	Non-RCT	No downgrading	Only one study meaning inconsistency is not applicable	No downgrading	Not Downgraded	None detected	⊕○○○ **Very low**
